# Microglia Control Neuronal Network Excitability via BDNF Signalling

**DOI:** 10.1155/2013/429815

**Published:** 2013-09-05

**Authors:** Francesco Ferrini, Yves De Koninck

**Affiliations:** ^1^Department of Veterinary Sciences, University of Turin, Grugliasco, 10095 Turin, Italy; ^2^Institut Universitaire en Santé Mentale de Québec, Québec, QC, Canada G1J 2G3; ^3^Department of Psychiatry and Neuroscience, Université Laval, Québec, QC, Canada G13 7P4

## Abstract

Microglia-neuron interactions play a crucial role in several neurological disorders characterized by altered neural network excitability, such as epilepsy and neuropathic pain. While a series of potential messengers have been postulated as substrates of the communication between microglia and neurons, including cytokines, purines, prostaglandins, and nitric oxide, the specific links between messengers, microglia, neuronal networks, and diseases have remained elusive. Brain-derived neurotrophic factor (BDNF) released by microglia emerges as an exception in this riddle. Here, we review the current knowledge on the role played by microglial BDNF in controlling neuronal excitability by causing disinhibition. The efforts made by different laboratories during the last decade have collectively provided a robust mechanistic paradigm which elucidates the mechanisms involved in the synthesis and release of BDNF from microglia, the downstream TrkB-mediated signals in neurons, and the biophysical mechanism by which disinhibition occurs, via the downregulation of the K^+^-Cl^−^ cotransporter KCC2, dysrupting Cl^−^homeostasis, and hence the strength of GABA_A_- and glycine receptor-mediated inhibition. The resulting altered network activity appears to explain several features of the associated pathologies. Targeting the molecular players involved in this canonical signaling pathway may lead to novel therapeutic approach for ameliorating a wide array of neural dysfunctions.

## 1. Introduction

Once simply considered as the “guardians” of the central nervous system (CNS), microglia have more recently emerged as key players in regulating neuronal network excitability. Indeed, physical and chemical alterations in the extracellular environment promote the synthesis and release of several microglia-derived molecules which, in turn, shape neuronal circuit function. The effects of such microglia-neuron interactions were found to be critical in the course of different central disorders, and in particular seminal studies provided significant evidence for a role of microglia in the pathogenesis of seizures (for review see [1, 2]) which was associated with increased glutamatergic transmission through the potentiation of NMDA receptor-mediated activity [3]. However, from a theoretical point of view, raising network excitability can be equally achieved through increasing excitatory inputs or removing inhibitory ones. In fact, unmasking silent interconnections can be better achieved through disinhibition than enhanced excitation. Furthermore, disinhibition has been shown as an upstream substrate of activity-dependent enhancement of excitation in several plasticity paradigms [4–7]. Thus, in addition to the glutamatergic hypothesis, it can be postulated that microglia alter neuronal excitability by affecting synaptic inhibition. This hypothesis has been explored during the last decade and the results of several investigations have uncovered molecular mechanisms underlying microglia-mediated disinhibition.

Synaptic inhibition in central neurons is mediated by *γ*-amino-butyric acid (GABA) and glycine (Gly) which activate ionic channels (GABA_A_R and GlyR) permeable to anions, namely, chloride (Cl^−^) and bicarbonate (HCO_3_^−^). Under physiological conditions, Cl^−^ flows inwardly and HCO_3_^−^ outwardly, along with their electrochemical gradient. Cl^−^ contribution is by far the more conspicuous, and, consequently, the reversal potential of GABA/glycine (EGABA/Egly) in adult neurons is set below the resting potential (Vr) near the Cl^−^ equilibrium (ECl). It follows that when GABA_A_R/GlyR are activated, Cl^−^ produces a net hyperpolarization. Although in principle correct, this brief summary of the ionic mechanisms of synaptic inhibition provides a quite static representation of GABA/Gly-mediated transmission and importantly does not take into account that, EGABA/EGly and Vr being only few millivolts apart, even a small change in anion concentrations may have a profound functional impact [8, 9]. In this respect, the critical variable is represented by the intracellular Cl^−^ concentration and the critical property is the capacity of the cell to maintain this concentration low. In the event that intracellular Cl^−^ rises, it follows that (i) EGABA/EGly shifts toward or beyond Vr; (ii) the Cl^−^ gradient across the membrane collapses; and (iii) the previously negligible depolarizing HCO_3_^−^ current becomes more relevant. On the whole, an increase in the intracellular Cl^−^ concentration weakens the strength of GABA/Gly-mediated inhibition or, in the extreme case, turns it into paradoxical excitation [10].

How do neurons control intracellular Cl^−^ concentration? Chloride homeostasis in cells is maintained by a group of membrane carriers known as cation-chloride cotransporters (CCCs [11, 12]). The K^+^-Cl^−^ cotransporter 2, KCC2, is the main CCC isoform expressed in central neurons [13, 14]. KCC2 extrudes Cl^−^ following the K^+^ gradient, and its activity typically maintains a low intracellular Cl^−^ concentration, which is the prerequisite for an effective GABA/Gly-mediated inhibition. Now, KCC2 activity is not static, but it can be profoundly modulated by different physiological or pathological challenges. The most spectacular example of such plasticity has been extensively described during development [14–16]. KCC2 is little expressed in prenatal and early postnatal brains, but during maturation it undergoes a developmental increase, which parallels the switch in GABA/Gly-mediated transmission from excitatory to inhibitory [14]. These mechanisms are thought not only to play a pivotal role in the activity-dependent development of central synapses during CNS maturation [17] but also to favor a proper wiring by triggering spontaneous rhythmic activity in motor networks [18] and to promote synaptic integration of new born neurons in those area of the brain in which adult neurogenesis occurs [19, 20]. On the other hand, reduction of KCC2 activity has been associated with several neurological diseases and conditions, originally epilepsy and neuropathic pain [10], and more recently motor spasticity [21], stress [22], and schizophrenia [23, 24]. Several lines of evidence accumulated during the last decade have indeed demonstrated that the increase in excitability in these pathological conditions can be largely explained by a loss of inhibition, and KCC2 has been recognized as a key molecular target underlying this loss [25, 26].

The findings in recent years that KCC2 can be dynamically modulated by several intercellular signaling pathways have been particularly interesting [4], the most prevalent being brain-derived neurotrophic factor (BDNF) signaling onto neuronal TrkB receptors [27–31]. Even more intriguing is the finding that, in certain conditions, BDNF in the CNS is not only released by neurons but also by microglia [32]. In this review we summarize and discuss the more relevant findings supporting the role of microglia in conditioning KCC2 function, as well as consequently inhibitory neurotransmission, through the release of BDNF. Several convergent findings uncover a canonical signaling mechanism by which the immune system can control neuronal network excitability by regulating the strength of inhibition.

## 2. Role of BDNF in the Control of KCC2 Function

BDNF is a neurotrophin with important functions in neuronal survival and differentiation. However, beyond its classical neurotrophic role, BDNF is directly involved in the control of neuronal activity and synaptic plasticity as a neuromodulator [33–35]. These functions are described in several areas of the CNS, such as hippocampus [36], cortex [37], amygdala [38], cerebellum [39], and spinal cord [34], and are involved in different forms of plasticity [40, 41]. Although initial studies mainly focused on glutamatergic synapses, the effects of BDNF on GABAergic transmission have lately received increasing attention [40]. Interestingly, early work on these effects performed in the rat hippocampus yielded a number of conflicting results, unveiling a more complex picture than expected. Indeed, in juvenile rodents BDNF was found to favor a substantial depression of GABAergic transmission via either pre- or postsynaptic mechanisms [42, 43]; conversely, studies in immature neurons showed an overall potentiating effect [44, 45]. To explain such a discrepancy, it was hypothesized that the effect of BDNF onto GABAergic transmission in hippocampal neurons might be developmentally regulated in parallel with the switch in GABAergic transmission from excitatory to inhibitory [46]. Thus, BDNF depresses GABAergic transmission in mature neurons when GABA is inhibitory and potentiates it in immature neurons when GABA is depolarizing, favoring activity-dependent synapse formation which has been relayed to GABA-mediated Ca^2+^ entry in developing neurons [37, 44, 46]. The fact that the changes in BDNF effects on GABA-mediated transmission are coincident with the developmental switch in GABAergic current polarity raised the question of whether BDNF has an effect on KCC2 function and/or expression. This was indeed demonstrated by Rivera and colleagues [29, 30]. The authors provided evidence that, in hippocampal slices, BDNF rapidly downregulates KCC2 expression through the BDNF preferred receptor TrkB (tyrosine kinase B receptor), thus reducing neuronal Cl^−^ extrusion capacity [29]. The effect required the activation of two downstream cascades involving src homology 2 domain containing transforming protein/FGF receptor substrate 2 (Shc/FRS-2) and phospholipase C *γ*- (PLC*γ*-) cAMP response element-binding protein signaling, respectively [30]. Interestingly, the activation of the Shc pathway alone was surprisingly found to promote the upregulation of KCC2, which might elegantly explain the opposite effects of BDNF across brain development based on the specific intracellular pathways involved [30]. One important point of this study is that the membrane level of KCC2 undergoes a fast turnover rate, and this turnover is accelerated by exogenous BDNF or by an increased neuronal activity during which BDNF is released [30]. A logical consequence is that such a fast regulation of KCC2 activity, which happens in few hours or less, is not compatible with the physiological time course required for altering gene expression, and a number of alternative mechanistic models have been proposed including protein phosphorylation, trafficking, and quaternary structure [47]. In particular, KCC2 activity and membrane localization seem to depend on the tyrosine phosphorylation level, and BDNF has been shown to promote KCC2 dephosphorylation, which in turn reduces surface protein expression [48]. Thus, KCC2 phosphorylation influences protein trafficking by either increasing endocytosis or reducing insertion [48]. Alternatively, KCC2 transport activity has been directly correlated with the capacity of the protein to form oligomers at the membrane level [49]. Thus, Cl^−^ extrusion capacity is improved if KCC2 is organized in oligomers, and an increased oligomers/monomers ratio parallels the KCC2 upregulation during development [49]. Interestingly, KCC2 clustering is strongly reduced in the presence of a point mutation on the KCC2 tyrosine phosphorylation site, suggesting that phosphorylation and oligomerization might simply be different parts of the same process controlling the transporter activity [50]. Finally, KCC2 activity can also be rapidly affected by the activation of the Ca^2+^-dependent protease calpain [11], and these pathways may be under the control of BDNF/TrkB signaling [51].

Altogether, these findings provided clear evidence that Cl^−^ homeostasis can be rapidly regulated by an extracellular signal, such as BDNF, thus inducing short- or long-term changes in neuronal activity that cannot be simply explained in terms of classical synaptic plasticity but rather as a novel form of “ionic plasticity” [16].

After the initial studies on the effects of BDNF on Cl^−^ homeostasis in CA1 pyramidal neuron of the hippocampus [29, 30], similar mechanisms were subsequently observed in different regions across the CNS, including the spinal dorsal horn [27] and ventral horn [21], the ventral tegmental area [52], the cortex [53, 54], and the cerebellum [39]. These findings attracted attention to the fact that BDNF may play a pivotal role as a regulator of neuronal Cl^−^ homeostasis in the brain and, by ricochet, of inhibition and hence neuronal network excitability.

## 3. Microglia Are a Central Source of BDNF

The expression of BDNF in synaptic vesicles and its synaptic release from different neuronal populations [34, 55] support the role of the neurotrophin in activity-dependent downregulation of KCC2 [30]. However, BDNF is not only expressed by neurons but is also found in astrocytes [56] and microglia [32]. Microglial BDNF was first shown in microglia cultures [57, 58] and soon confirmed in different regions of the CNS during the course of various neurological disorders, such as viral encephalitis [59], traumatic injury [60, 61], ischemia [62], multiple sclerosis [63], Parkinson's disease [64], neuropathic pain [27], and spasticity [21]. That microglia are a potential source of BDNF is a crucial point to predict the role of the neurotrophin in neurological disorders. Indeed, microglia primary function is to sense and react to alterations of the extracellular milieu with a protective and defensive role. In the presence of factors signaling potentially harmful, microglia undergo morphological and functional alterations collectively identified under the term of “microglia activation,” and, depending on the signaling pathways involved, this process may lead to secretion of specific messengers, including BDNF [65]. Once released, the neurotrophin in turn sculpts neuronal circuit excitability via the signaling cascade described above.

The synthesis and release of BDNF in microglia appear to be tightly associated with the purinergic receptor P2X4R [65–67]. Purinergic receptors are endogenously activated by ATP (Adenosine-5′-triphosphate), which is typically stored in the cytoplasm of neuronal and nonneuronal cells and released in the extracellular space following tissue damage [68]. Alternatively, ATP may be released by neurons [69] or astrocytes [70]. Microglia sense extracellular ATP trough different types of purinergic receptors [68], such as P2Y12Rs, which can detect tiny gradient of extracellular ATP and promote microglia migration [71, 72], or P2X7Rs, which trigger morphological changes in microglia from a resting to an activated state [73]. Microglial P2X4Rs, instead, do not appear to be involved in the morphological alterations leading to the activated phenotype, but rather their involvement is a functional consequence of microglia activation [65]. Indeed, P2X4Rs are normally expressed at negligible levels in resting microglia, and they need to be upregulated to promote BDNF synthesis and release [65]. Which external factors are involved in the upregulation of P2X4R in activated microglia is still a matter of debate. Chemokines released from injured neurons, such as CCL2 and CCL21, have been regarded as potential inductors of P2X4R expression [74, 75]. In particular, CCL21 application *in vivo* and *in vitro* strongly promoted P2X4R upregulation in spinal microglia [74]. Interestingly, in both CCL21 [74] and P2X4R [65] deficient mice microglia activation is not compromised, which implies a mechanistic separation between the morphological changes and the subsequent downstream effects. Also CCL2, which instead plays an important role in microglia activation after injury [76, 77], has been suggested to participate in the P2X4R upregulation process; however, CCL2 does not seem involved in *de novo* expression of the protein, but rather it has been suggested to promote P2X4R trafficking from intracellular stores to the cell membrane [75]. Finally, a few nonneuronal endogenous molecules have been also identified as potential inductors of P2X4R in microglia, namely, the proinflammatory cytokines INF-*γ* [78], the mast cell-derived tryptase activated PAR2 [79], and fibronectin, a component of the extracellular matrix [80, 81]. At the nuclear level, the interferon regulatory factor 8 (IRF8) has been recently proposed as a key transcription factor involved in the upregulation of P2X4Rs in activated microglia [82].

Once upregulated, P2X4Rs can efficiently respond to extracellular fluctuation in ATP concentration and initiates the intracellular cascade leading to BDNF synthesis and release. Being particularly highly Ca^2+^ permeable, P2X4 channels cause a significant Ca^2+^ influx and the downstream activation of Ca^2+^-dependent intracellular pathways, among which the phosphorylation of p38 MAP kinase, which is directly involved in the synthesis and release of BDNF [67]. In addition Ca^2+^ influx through P2X4Rs is also necessary to directly facilitate the release of BDNF by acting on the vesicle-releasing machinery, which is typically an NSF-attachment protein-(SNARE-) mediated exocytosis [67]. Alternative pathways (i.e., ERK1/2) have been also suggested to promote BDNF synthesis in cultured microglia [83, 84]; however, these hypotheses need to be properly confirmed *in vivo*.

## 4. The Special Case of Neuropathic Pain

Based on the findings outlined above, the following conclusions can be drawn: (1) various extracellular signals may activate microglia and upregulate P2X4Rs; (2) P2X4R activation triggers the release of BDNF from microglia; (3) BDNF-TrKB signaling alters KCC2 function leading to a reduced Cl^−^ extrusion capacity which dampens GABA_A_R/GlyR mediated inhibition. Assuming that all these events happen in sequence, one should expect that microglia, under certain functional states, influence synaptic inhibition. This is indeed the case of neuropathic pain [66]. Nociceptive transmission is normally conveyed to higher centers through spinal nociceptive pathways. In the most simple configuration, this involves peripheral neurons located in the dorsal root ganglia, which contact second-order neurons in the spinal dorsal horn, and a spinal projection neurons which transmits the information to the thalamus. In the spinal dorsal horn, pain transmission is controlled by a network of local inhibitory interneurons which assure the separation of nociceptive sensory pathways from nonnociceptive sensory pathways by releasing GABA and Gly [85]. Indeed, a spinal administration of GABA_A_R or GlyR antagonists induces tactile allodynia [86, 87], a clinical condition in which innocuous stimuli are perceived as painful. Tactile allodynia is a classical symptom of neuropathic pain and indicates an erroneous encoding of low threshold stimuli through the nociceptive channel. Several causal events have been postulated to promote spinal disinhibition, including presynaptic mechanisms affecting the amount of transmitter released and intracellular pathways regulating postsynaptic GABA and glycine receptor function/expression [7]. In our laboratory, we found that altered Cl^−^ homeostasis in the superficial spinal dorsal horn appears as a key mechanism underlying neuropathic pain symptoms [88] and that this alteration results from the release of BDNF from microglia [27]. Microglia had already been implicated in the pathogenesis of neuropathic pain [89–91], and in particular the upregulation of P2X4Rs in microglia was early identified as a crucial step in the central sensitization process [92]. P2X4Rs are in fact necessary for the development of mechanical allodynia after nerve injury and are required for the release of BDNF from microglia [65, 67]. BDNF in turn binds TrkB receptors in neurons of the superficial dorsal horn, thus compromising KCC2 function and altering Cl^−^ homeostasis [27]. Blocking the microglia-to-neuron cascade at any level reverses established allodynia in neuropathic animals by restoring spinal inhibitory GABAergic/glycinergic transmission [27, 28]. Subsequent studies have provided additional evidence that this form of spinal disinhibition happens in a different model of pathological pain, such as spinal cord injury [93], diabetes-induced neuropathy [94], and orofacial pain [95]. Moreover, we have very recently shown that the pain hypersensitivity induced by morphine (better known as morphine-induced hyperalgesia) is mediated by the same P2X4Rs-BDNF-TrkB-KCC2 cascade, thus recapitulating the sequence of events described in neuropathic pain [28]. In the latter study, we used a transgenic mouse in which BDNF expression was genetically ablated in microglia only, and we showed that, without microglial BDNF, morphine hyperalgesia does not take place. The involvement of spinal microglia in this specific form of hypersensitivity is due to the expression of opioid receptors in microglia [84] whose activation promotes P2X4Rs [28]. In turn, morphine appears to act on microglia via a nonopioid receptor-dependent pathway to enable BDNF release upon P2X4Rs activation [28].

In conclusion, ten years of investigations on the spinal mechanisms of nociceptive transmission have provided compelling evidence that neuropathic pain critically depends on microglia-to-neuron signals which alter GABA/glycine-mediated inhibition.

## 5. Microglia-BDNF-KCC2 Signaling in the Pathogenesis of Multiple Neurological Conditions

The microglia-to-neuron communication discovered in the dorsal horn of the spinal cord can be virtually replicated in all those regions of the CNS where functional TrkB receptors are expressed and may play a role in the development of multiple central disorders [10]. Accumulating evidence in recent years supports this hypothesis.

In the spinal motor system, a TrkB-KCC2 interaction has been described in motoneurons following spinal cord injury [21]. Here, the reduced Cl^−^ extrusion capacity due to the downregulation of KCC2 was associated with hyperreflexia and spasticity, a clinical condition burdening a large number of patients with spinal trauma [96]. The authors did not investigated the origin of BDNF in their model; however microglia are clearly involved in the pathophysiology resulting from spinal cord injury [97], and a role for microglial P2X4Rs has also been envisaged [98], suggesting a microglial BDNF link.

In the brain, alterations in Cl^−^ homeostasis have been shown to underlay epilepsy in animals and humans [99]. Based on experiments *in vitro* on hippocampal slices [29, 30], TrkB-KCC2 signaling was proposed as the molecular mechanism underlying hyperexcitability in epilepsy [30]. In this model, however, KCC2 downregulation was shown to be activity dependent, thus implying a neuronal source of BDNF whose release is directly related to the level of network excitability. On the other hand, epilepsy has multiple etiologies and might develop in different brain areas. A role for microglia can be therefore predicted in those pathological conditions which imply a neuronal damage and the subsequent reorganization of synaptic function, as in the case of a traumatic event [100]. Indeed, a TrkB-dependent downregulation of KCC2 has also been described in traumatic brain injury [101], a condition in which neuronal death and inflammation clearly promote the activation of microglia [102]. Interestingly, in animal models of traumatic brain injury, microglia were found to express P2X4Rs and phosphorylated p38 [103, 104], which is known to be the main upstream signal for BDNF synthesis and release in microglia [67].

Finally, a BDNF-mediated impairment of Cl^−^ homeostasis has been shown to underlie the central mechanisms of opiate dependence in the ventral tegmental area (VTA) [52]. Although the main source of BDNF remains here elusive, chronic exposure to opioids is known to activate microglia and to induce the synthesis of BDNF [28, 84], which, in turn, impairs Cl^−^ homeostasis in central neurons [28]. In addition, it has recently been described that functional modifications in microglia are involved in mechanisms of opiate dependence in the nucleus accumbens where the early exposure to morphine in young rats was shown to influence drug-seeking behavior in adulthood increasing the risk of drug-induced reinstatement [105].

Taken together, these evidence indicate that BDNF-TrkB signaling drives disinhibition by targeting KCC2 function. Such an effect does not directly depend on the source of BDNF (neuron, astrocytes, or microglia) but rather on the intracellular pathways linking TrkB to KCC2 [29]. This is exemplified in immature neurons where BDNF-TrkB signaling, rather than causing KCC2 downregulation, stimulates the synthesis of KCC2 and favors the developmental switch of GABAergic transmission from excitatory to inhibitory [106]. In contrast, microglial BDNF has gained special attention as underlying neurological diseases in adult tissue, and this is mainly due to the specific role played by microglia in the CNS. Indeed, microglia-driven disinhibition via BDNF-TrkB signaling can be regarded as a peculiar consequence of microglial reaction to injury or to certain pharmacological treatments, potentially occurring in different areas of the CNS. The “pathological” consequences of such process are usually dramatic, leading for instance to an altered nociceptive behavior or to seizure. It remains enigmatic what the normal “physiological” meaning of the release of BDNF from microglia and the subsequent downregulation of KCC2 is. The primary role of microglia is in fact to react in response to a variety of external challenges supposedly with the aim of protecting neurons. In this context, the release of BDNF can be considered as a part of a neuroprotective strategy, being neurotrophins classically involved in neuronal survival process [107]. A neuroprotective and reparative role for microglial BDNF has indeed been postulated during the course of encephalitis [59], brain ischemia [62], and traumatic injury [60]. Interestingly, the posttraumatic loss of KCC2 in mature neurons induced by BDNF and the subsequent GABA-mediated depolarization was found necessary for neuronal survival of injured neurons, a mechanism which is strongly reminiscent of the trophic effect of excitatory GABA during CNS development [101]. In contrast, the central inflammatory reaction in the spinal dorsal horn following peripheral nerve injury appears to be substantially maladaptive and detrimental. Indeed, the activation of spinal microglia following nerve injury produces a release of BDNF onto spinal neurons which are not directly injured. The main effect of microglial activation in this case is thus the suppression of spinal inhibition and the activation of nociceptive pathways, leading to the clinical development of neuropathic pain [27]. Microglia therefore appear as an ambiguous actor, in some cases protective and in other cases having deleterious actions [108]. An accurate prediction of the balance between neuroprotection and neurotoxicity appears thus important to understand how microglia intervene in diseases to develop appropriate therapeutic strategies.

**Figure 1:**
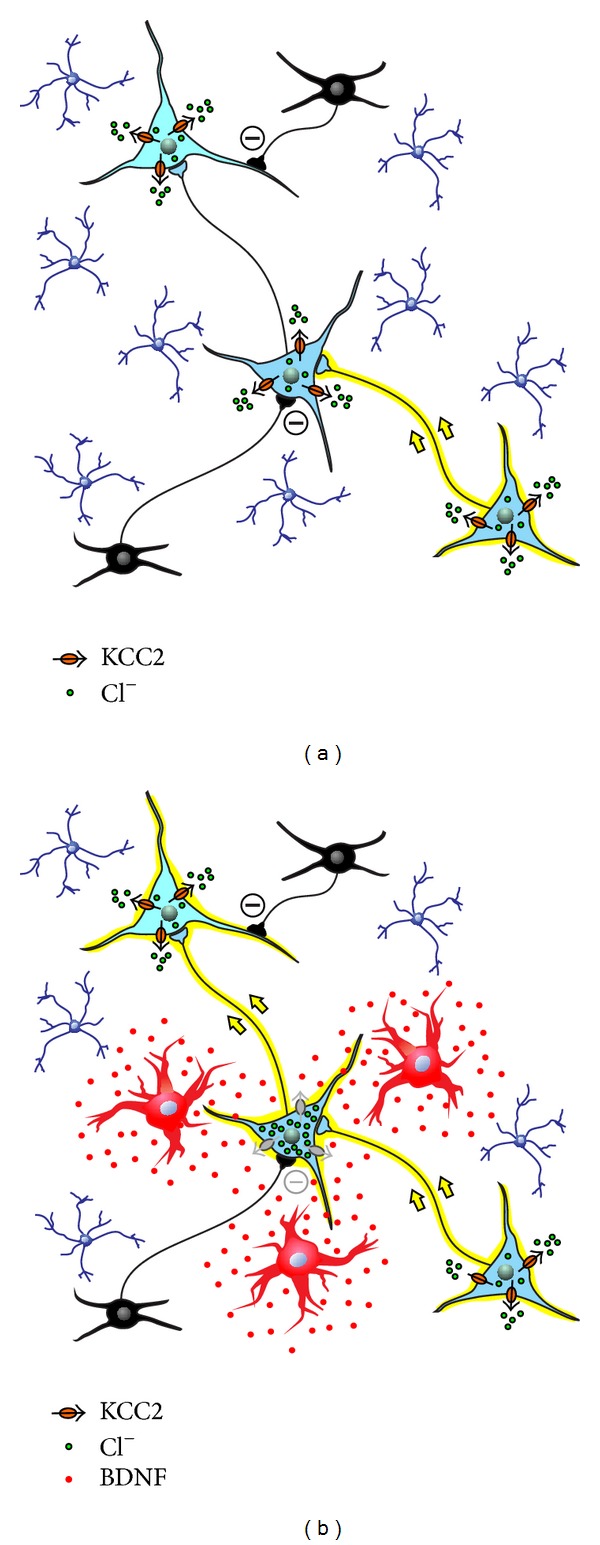
Microglia control neuronal network excitability via secretion of BDNF. The left panel illustrates a schematic neuronal network in the mature CNS under normal conditions: microglia (blue ramified cells) are in their resting state; small inhibitory interneurons release GABA or Gly to repress the flow of signals across the network; normal KCC2 activity extrudes Cl^−^ (black arrows) to maintain the Cl^−^ gradient, and, consequently, Cl^−^ flows in through GABA_A_R/GlyR channels to inhibit activity. The right panel illustrates the same network after an external event has induced microglial activation (red cells) and the release of microglial BDNF: BDNF-TrkB signaling causes downregulation of KCC2; Cl^−^ accumulates in neurons and the Cl^−^ gradient collapses; GABA_A_R/GlyR-mediated inhibition is less effective in controlling neuronal firing, and previously silent neuronal pathways are unmasked (yellow arrows).

Yet, regardless of the positive or negative outcome of microglia action on neuronal survival and repair, the activation of the P2X4R-BDNF-TrkB-KCC2 cascade allows microglia to critically control network excitability and to unmask hidden neuronal circuits that are normally kept silent by the physiological Cl^−^-mediated inhibition (Figure 1) [109].

## 6. Future Directions

The signaling cascade described in this review represents a molecular substrate underlying the mechanism by which microglia target GABAergic/glycinergic neurotransmission. However, it is likely that BDNF released from microglia also challenge network excitability by mechanisms other than KCC2. In particular, BDNF-TrkB signaling also targets NMDA receptors [65, 110], and microglial BDNF has been suggested to underlie certain forms of pathological pain via the activation of spinal NMDA [111]. The outcome of both KCC2 downregulation and NMDA potentiation is an overall increase in network excitability. This raises the question of whether modulationsof KCC2 and NMDA functions are independent processes or are reciprocally connected. Several lines of evidence support the latter hypothesis [112, 113], and future investigations are encouraged to further explore such interactions in different neurological disorders. In addition to BDNF, microglia are known to directly or indirectly modulate synaptic transmission through the release of tens of other different molecules [114]. Most of past studies have differently focused on the effect of these molecules in the modulation of glutamatergic transmission. In this respect, a role has been described for cytokines [115], glycine [116], NMDA receptor agonists [117], adenosine [118], and ATP [119]. On the other hand, a growing body of studies reported that cytokines might also directly modulate GABAergic transmission [115]: interleukin 1*β* was found to depress GABA release in a model of autoimmune encephalitis [120] and to potentiate GABAergic transmission in CA1 [121] or in hypothalamic neurons [122]; both interleukin 6 and interleukin 1*β* were seen to reduce GABA- and Gly-mediated currents in the spinal dorsal horn [123]; tumor necrosis factor *α* was shown to promote GABA_A_R endocytosis in hippocampal neurons thus weakening the inhibitory synaptic strength [124]. In addition, microglia also produce lipophilic gaseous molecules, such as nitric oxide [125–127], and lipidic inflammatory mediators, such as prostaglandins [127, 128]. Interestingly, prostaglandin E2 directly suppresses glycine-mediated transmission in the spinal dorsal horn, a mechanism centrally involved in the development of inflammatory pain [129, 130]. Deeper insights into the role played by each of these messengers in normal and pathological conditions are required to improve our understanding of the role of microglia-to-neuron communication.

Yet, the effects reported in different studies for most of these microglia-derived molecules are often quite dissimilar and critically influenced by the experimental paradigms, drug concentrations, and neuronal populations considered [114]. In addition, the mechanisms leading to the release of specific molecules, as well as the molecular pathways activated in neurons, are still poorly understood, making it difficult to draw a coherent picture for their role in synaptic transmission. Conversely, the P2X4R-BDNF-TrkB-KCC2 cascade described here appears to connect altered extracellular conditions with microglia activation, neuronal excitability, and eventually the development of a pathological behavior. Collectively, these findings open new important therapeutic avenues for the control of neuropathic pain [25] and epilepsy [99]. Yet, many questions are left unanswered and need to be addressed to better delineate the range of applications for an effective microglia-targeted therapeutic strategy; in particular: which neurological disorders are associated with a microglia-driven loss of inhibition? In which brain areas? Do all microglia have the same potential to synthesize and release BDNF when exposed to a given extracellular challenge? Or, instead, are microglia a heterogeneous population with multiple phenotypes playing different roles in different CNS areas and in different pathological states?

Tackling the multiform universe of microglia-neuron interactions and understanding the underlying molecular pathways offer the opportunity to identify specific biomarkers for neurological disorders and potential targets for novel therapeutic approaches.
